# Thrombus formation in neonates and early infants undergoing congenital heart surgery

**DOI:** 10.1016/j.ijcchd.2025.100617

**Published:** 2025-08-27

**Authors:** Muneaki Matsubara, Alessandra Poppe, Thibault Schaeffer, Jonas Palm, Teresa Lemmen, Paul Philipp Heinisch, Nicole Piber, Andrea Amici, Alfred Hager, Peter Ewert, Jürgen Hörer, Masamichi Ono

**Affiliations:** a Department of Congenital and Pediatric Heart Surgery, Technical University Munich, German Heart Center, Munich, Germany; b Division of Congenital and Pediatric Heart Surgery, University Hospital of Munich, Ludwig-Maximilians-Universität München, Munich, Germany; c Europäisches Kinderherzzentrum München, Munich, Germany; d Department of Congenital Heart Disease and Pediatric Cardiology, Technical University Munich, German Heart Center, Munich, Germany; e Department of Cardiovascular Surgery, Technical University Munich, German Heart Center, Munich, Germany

**Keywords:** Thrombus, Neonate, Tricuspid valve repair, Norwood procedure

## Abstract

**Objective:**

This study evaluated thrombus formation and its impact on outcomes in neonates and early infants undergoing congenital heart surgery.

**Methods:**

Neonates and early infants (≤90 days) undergoing congenital heart surgery with cardiopulmonary bypass from 2001 to 2024 were analyzed. Thrombi were detected by transthoracic echocardiography and cardiac catheterization.

**Results:**

Among 2331 patients, 170 (7.3 %) developed thrombi during hospitalization. Median age at surgery and time to thrombus detection in affected patients were 12 (interquartile range: 7–34) and 7 (interquartile range: 3–15) days, respectively. Among surgical procedures performed in at least 10 patients, thrombi were most frequently observed following tricuspid valve repair (28.6 %), followed by arterial switch operation, ventricular septal defect closure, and aortic arch repair (15.8 %). The most common thrombus location was the superior vena cava in 61 patients, followed by the inferior vena cava in 33, the aorta in 31, and the right atrium in 21 patients. Additional surgical interventions were required in 28 patients. The length of hospital stay was significantly longer in patients with thrombi (27 vs. 15 days, p < 0.001). Independent risk factors for thrombus formation included preoperative cardiopulmonary resuscitation (odds ratio: 2.037, p = 0.001), tricuspid valve repair (odds ratio: 6.206, p < 0.001), and Norwood procedure (odds ratio: 1.558, p = 0.027).

**Conclusions:**

The incidence of thrombus formation was 7.3 % in neonates and early infants undergoing congenital heart surgery. Thrombus was most frequently observed in the superior vena cava and resulted in prolonged hospitalization. Preoperative cardiopulmonary resuscitation, tricuspid valve repair, and Norwood procedures carried the highest thrombotic risk.

## Highlights

• Thrombus incidence was 7.3 % in neonates undergoing congenital heart surgery.
• Tricuspid valve repair carried the highest thrombotic risk (28.6 % incidence).
• Median thrombus detection time was 7 days, emphasizing need for close monitoring.

## Abbreviations and acronyms

APTT: activated partial thromboplastin time
CHD: congenital heart disease
CPB: cardiopulmonary bypass
CPR: cardiopulmonary resuscitation
ECMO: extra-corporeal membrane oxygenation
ICU: intensive care unit
IQR: interquartile ranges
IVC: inferior vena cava
OR: odds ratio
POD: postoperative days
STAT: The Society of Thoracic Surgeons-European Association for Cardio-Thoracic Surgery
SVC: superior vena cava

## 1 Introduction

Neonates and early infants with congenital heart disease (CHD) often undergo corrective or palliative cardiac surgical procedures and are at risk for significant morbidity and mortality [1]. Despite its clinical importance, data on thrombosis following cardiac surgery in neonates and early infants remain limited. Neonates and early infants represent a particularly high-risk population for thrombotic complications following cardiac surgery [2]. Cardiac surgery in these patients is associated with disruption of blood flow, platelet dysfunction and activation, inflammation, and blood hypercoagulability, all of which contribute to thrombus formation. The immature coagulation system in neonates exhibits reduced antithrombotic capacity and unpredictable responses to anticoagulation [[3], [4], [5]]. Additionally, many congenital heart lesions require shunt-dependent circulation, which is inherently prone to thrombotic occlusion. The incidence of postoperative thrombosis in children with CHD ranges widely, from 5 % to 33 %, with infants younger than one year at particularly high risk [6,7]. However, comprehensive data specifically addressing thrombotic complications in neonates and early infants undergoing cardiac surgery are scarce.

Our study aims to evaluate the incidence of postoperative thrombus formation in neonates and early infants who underwent cardiac surgery, assess its impact on mortality, and identify predisposing risk factors for thrombosis.

## 2 Methods

### 2.1 Data availability

The data underlying this article will be shared by the corresponding author upon reasonable request to protect patient confidentiality.

### Ethical statement

The study was approved by the Institutional Review Board of the Technical University of Munich (approval number 2025-232-S-NP, approved May 5, 2025). The requirement for individual patient consent was waived due to the retrospective design and patient privacy rights were observed.

### 2.2 Patients and data collection

A single-center retrosvpective study was conducted to review all neonates and early infants (≤90 days) with congenital heart disease who underwent palliative or corrective cardiac surgery with cardiopulmonary bypass (CPB) at the German Heart Center Munich between January 2001 and January 2024. Patients undergoing surgery without CPB were excluded from the analysis. Medical records were retrospectively reviewed for demographics, preoperative echocardiographic findings, procedural details, thrombus formation characteristics, and postoperative outcomes. The follow-up period was defined as the interval between congenital heart surgery and the most recent clinical examination, censored at death for deceased patients. Procedural risks were stratified using the Society of Thoracic Surgeons-European Association for Cardio-Thoracic Surgery (STAT) mortality score ranging from 1 to 5 [8].

### 2.3 Surgical techniques and perioperative management

Cardiac surgery was performed using standard CPB with bicaval venous and ascending aortic arterial cannulation. Cardiac arrest was achieved with antegrade Bretschneider's cardioplegic solution (20 ml/kg). Selective cerebral cannulation was performed for patients requiring aortic arch reconstruction. Modified ultrafiltration was routinely performed after weaning from CPB. Patients were transferred to the intensive care unit (ICU) intubated and extubated when hemodynamically stable. Postoperative thrombosis prophylaxis was standardized with unfractionated heparin administered intravenously at 5000 IU/m^2^ body surface area/day, targeting an activated partial thromboplastin time (APTT) of 60 s. This regimen was maintained until central venous line removal. Subsequently, patients with shunt diameter ≤4 mm received acetylsalicylic acid (3–5 mg/kg/day), while those with shunt diameter >4 mm received no further anticoagulation.

### 2.4 Detection of thrombus

Postoperative thrombus was primarily detected using transthoracic echocardiography, with angiography employed in select cases. Echocardiographic examinations were performed using a Vingmed ultrasonographic system (GE Vingmed Ultrasound AS; Strandpromenaden, Horten, Norway) equipped with 5.0-, 3.5-, or 2.5-MHz phased-array transducers. Cardiac catheterization and angiography were conducted for patients with problematic hemodynamics, including pulmonary artery angiography, aortography, and systemic venous angiography. Thrombus was defined as any localized echogenic mass detected within the heart or extracardiac locations adjacent to the heart. Thrombi located outside blood vessels, including those removed from the chest cavity, airway, or thoracic drainage tubes, were excluded. Thrombi associated with extracorporeal membrane oxygenation (ECMO) circuits were also excluded from analysis.

### 2.5 Statistical analysis

Categorical variables were expressed as numbers and percentages, and continuous variables as medians with interquartile ranges (IQR). Chi-squared tests were utilized for categorical variables. For continuous variables, Student's t-tests were applied for normally distributed data and Mann-Whitney U-tests for non-normally distributed data, as determined by Levene's test. Survival was calculated using the Kaplan-Meier method, with group differences determined by log-rank test. Risk factors for thrombus formation and mortality were identified using logistic regression models, with variables with p < 0.1 in the univariate analysis included in the multivariate model. All analyses were performed using SPSS version 28.0 (IBM, Ehningen, Germany) and R-statistical software (R Foundation for Statistical Computing, Vienna, Austria).

## 3 Results

### 3.1 Patient characteristics with and without thrombus formation

A total of 2331 neonates and early infants underwent palliative or corrective cardiac surgery during the study period. Thrombus formation was observed in 170 patients (7.3 %) during hospitalization. Patient characteristics are shown in Table 1. Patients with thrombus formation demonstrated significantly higher incidences of previous cardiopulmonary resuscitation (CPR) (16.5 % vs. 8.0 %, p < 0.001) and preoperative catecholamine administration (7.1 % vs. 3.8 %, p = 0.039). Additionally, they had longer CPB time (median 112 vs. 107 min, p = 0.003) and prolonged hospital stay (median 27 vs. 15 days, p < 0.001) compared to patients without thrombus formation.

**Table 1:** Baseline and operative characteristics of patients with and without thrombus.

Variable: N (%) or median (IQR)	All patients	Thrombus (+)	Thrombus (−)	p-value
Number of patients	2331	170 (7.3)	2161 (92.7)	
Female sex	903 (38.7)	62 (36.5)	841 (38.9)	0.528
Genetic anomalies	218 (9.9)	17 (7.8)	201 (9.9)	0.965
Extracardiac anomalies	360 (16.4)	26 (15.3)	334 (16.5)	0.689
Heterotaxy syndrome	49 (2.2)	2 (1.2)	47 (2.3)	0.332
Previous CPR	190 (8.7)	28 (16.5)	162 (8.0)	**<0.001**
Preoperative cathecholamine	89 (4.1)	12 (7.1)	77 (3.8)	**0.039**
Endocarditis	12 (0.5)	1 (0.6)	11 (0.5)	0.938
Age at procedures (days)	12 (7–39)	12 (7–34)	12 (7–39)	0.286
Weight at procedures (kg)	3.4 (3.0–3.8)	3.3 (2.9–3.7)	3.4 (3.0–3.8)	0.772
OP in naonates	1649 (70.7)	127 (74.4)	1522 (70.4)	0.238
OP in infants	682 (29.3)	43 (25.3)	639 (29.6)	0.238
Reoperation	338 (15.4)	42 (24.9)	296 (14.6)	**<0.001**
STAT category 4 or 5	1276 (54.7)	98 (57.6)	1178 (54.5)	0.429
CPB time (min)	108 (68–140)	112 (81–146)	107 (67–139)	**0.003**
Hospital stay (days)	15 (9–27)	27 (16–46)	15 (9–25)	**<0.001**

IQR: interquartile ranges, CPR: cardiopulmonary resuscitation, OP: operation, STAT: The Society of Thoracic Surgeons-European Association for Cardio-Thoracic Surgery, CPB: cardiopulmonary bypass.

### 3.2 Thrombus incidence according to procedural complexity

Thrombus incidence was similar between high-risk (STAT 4–5) and low-risk (STAT 1–3) procedures (7.7 % vs 6.8 %, p = 0.429). After adjustment for age, weight, CPB time, preoperative CPR, and catecholamine use, STAT category was not independently associated with thrombus formation (p = 0.785).

### 3.3 Incidence of thrombus formation according to the type of procedure

Procedures with the highest rates of thrombus formation are listed in Table 2. Results were stratified based on statistical considerations: procedures performed in at least 10 patients and those performed in fewer than 10 patients. Among procedures with more than 10 cases, the highest thrombus formation rates were observed in patients who underwent tricuspid valve repair (28.6 %), followed by arterial switch operation, ventricular septal defect closure, and aortic arch repair (15.8 %), and complete atrioventricular septal defect repair (13.3 %). For procedures with fewer than 10 cases, the highest thrombus formation rates were observed following mitral valve replacement (60.0 %), supravalvular aortic stenosis repair (33.3 %), and aortic valve replacement (20.0 %).

**Table 2:** Risk for ECMO implantation regarding operative procedure.

Variable: N (%)	All patients	Patients with Thrombus	p-value
Number of patients	2331	170 (7.3)	
Surgical procedures (>10)			
Tricuspid valve repair	21 (0.9)	6 (28.6)	**<0.001**
ASO + VSD + Aortic arch	19 (0.8)	3 (15.8)	0.153
CAVSD repair	30 (1.3)	4 (13.3)	0.200
Rastelli procedure	16 (0.7)	2 (12.5)	0.422
RVOT conduit exchange	16 (0.7)	2 (12.5)	0.422
Norwood-type procedure	389 (16.7)	41 (10.5)	**0.007**
BCPS	67 (2.9)	6 (9.0)	0.595
TOF repair with TAP	35 (1.5)	3 (8.6)	0.769
Aortopulmonary shunt	294 (12.6)	22 (7.5)	0.893
ASO for TGA	345 (14.8)	21 (6.1)	0.351
TAPVC repair	93 (4.0)	6 (6.5)	0.750
CoA repair	47 (2.0)	3 (6.4)	0.808
TAC repair	50 (2.1)	3 (6.0)	0.722
Aortic arch repair	138 (5.9)	8 (5.8)	0.486
ASO + VSD	91 (3.9)	5 (5.5)	0.501
VSD closure	155 (6.6)	7 (4.5)	0.169
CoA + VSD	48 (2.1)	2 (4.2)	0.400
ASD closure	26 (1.1)	1 (3.8)	0.497
Aortic arch repair + VSD	109 (4.7)	3 (2.8)	0.062
Ross	11 (0.5)	0 (0.0)	0.351
Mitral valve repair	15 (0.6)	0 (0.0)	0.276
TOF repair non TAP	29 (1.2)	0 (0.0)	0.129
Coronary procedure	17 (0.7)	0 (0.0)	0.246
Surgical procedures (<10)			
Mitral valve replacement	5 (0.2)	3 (60.0)	**<0.001**
Supra AS repair	6 (0.3)	2 (33.3)	**0.014**
Aortic vale replacement	5 (0.2)	1 (20.0)	0.274
TAC + IAA	6 (0.3)	1 (16.7)	0.377
Ross-Konno	7 (0.3)	1 (14.3)	0.476

ECMO: extra-corporeal membrane oxygenation, ASO: Arterial switch operation, VSD: Ventricular Septal Defect, RVOT: Right ventricular outflow tract, BCPS: Bi-directional cavo-pulmonary shunt, TOF: Tetralogy of Fallot, TAP: Tricuspid annuloplasty, TGA: Transposition of the great arteries, TAPVC: Total anomalous pulmonary venous connection, CoA: Coarctation of the aorta, TAC: Truncus arteriosus communis, AS: Aortic stenosis, IAA: Interrupted aortic arch.

### 3.4 Perioperative data in patients with thrombus formation

**Fig. 1:**
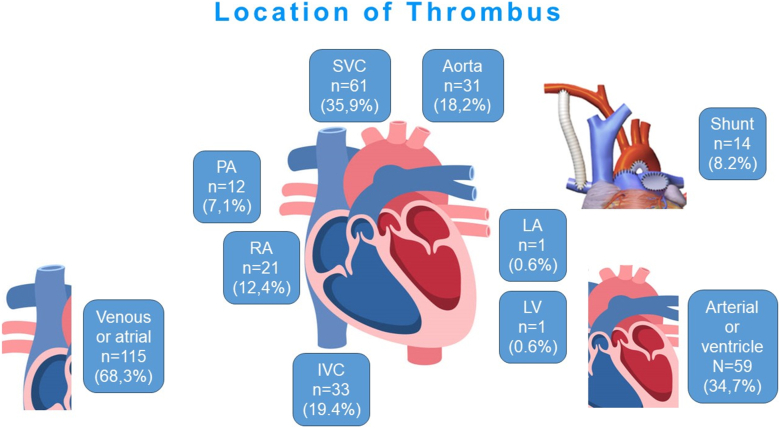
Location of thrombus.

Perioperative variables and hospital data for patients with thrombus formation are presented in Table 3. In patients who developed thrombus formation, median age at surgery and time to thrombus detection from surgery were 12 (IQR: 7–34) days and 7 (IQR: 3–15) days, respectively. Intrathoracic venous and atrial thrombi were identified in115 cases (68.3 %), and intrathoracic arterial or ventricular thrombi in 59 cases (34.7 %), including shunt thrombus in 14 cases (8.2 %, Fig. 1). The most common location was the superior vena cava in 61 patients, followed by the inferior vena cava in 33, the aorta in 31, and the right atrium in 21 patients. Thrombolytic therapy was administered to all patients, utilizing tissue plasminogen activators in 56 patients, high-dose heparin in 71 patients, or warfarin in 6 patients. Additional surgical interventions were required in 28 patients.

**Table 3:** Characteristics of patients with thrombus.

Variable: N (%) or median (IQR)	All (n = 170)
Age at operation (days)	12 (7–34)
Thrombus formation	
Median duration after the surgery (days)	7 (3–15)
Location of the thrombus	
Venous and atrial thrombus	115 (68.3)
Superior vena cava	61 (35.9)
Right atrium	21 (12.4)
Inferior vena cava	33 (19.4)
Left atrium	1 (0.6)
Arterial or ventricle thrombus	59 (34.7)
Aorta	31 (18.2)
Aortic valve	1 (0.6)
Pulmonary artery	12 (7.1)
Left ventricle	1 (0.6)
Aortopulmonary shunt	14 (8.2)
Therapy	
Medication	170 (100.0)
Surgery	28 (16.5)
In-hospital mortality	29 (17.1)
Median duration after initial operation (days)	14 (6–23)

IQR: interquartile ranges.

### 3.5 Postoperative coagulation data

**Fig. 2:**
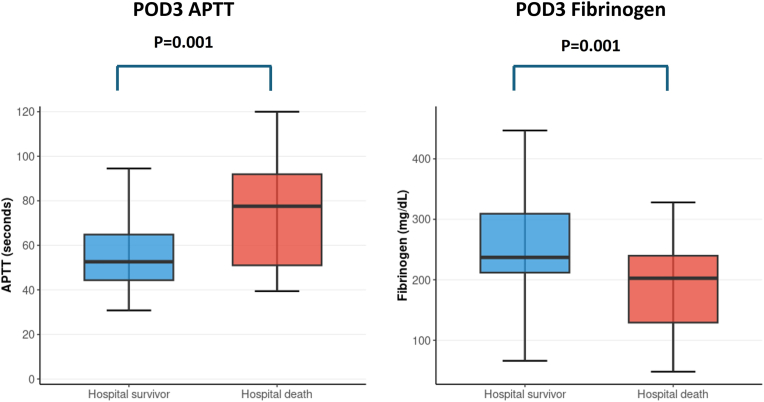
Coagulation parameters on postoperative day 3 by hospital outcome.

Coagulation parameters were analyzed in 170 patients with thrombus formation, including 141 hospital survivors and 29 patients who died during hospitalization. APTT, prothrombin time international normalized ratio, antithrombin III, and fibrinogen levels were evaluated preoperatively and on postoperative days (POD) 1, 3, 7, and 14. Significant differences between hospital survivors and non-survivors emerged on POD 3 (Fig. 2). Non-survivors demonstrated prolonged APTT (75.8 ± 26.0 vs. 60.0 ± 22.3 s, p < 0.001) and lower fibrinogen levels (197.2 ± 97.7 vs. 259.2 ± 101.4 mg/dL, p < 0.001) compared to survivors.

### 3.6 Risk factors analysis

**Fig. 3:**
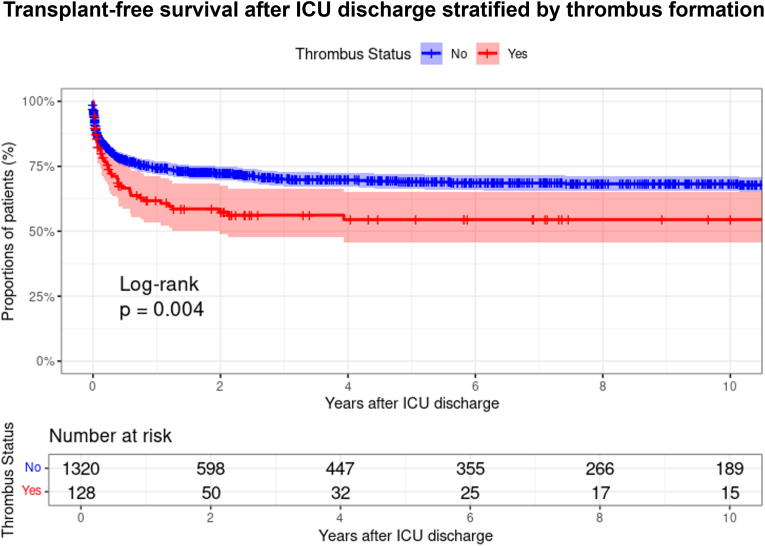
Transplant-free survival after intensive care unit discharge stratified by thrombus formation.

Logistic regression analysis results are presented in Table 4a, Table 4b. Independent risk factors for thrombus formation were identified as tricuspid valve repair (odds ratio (OR): 6.206; p < 0.001), Norwood procedure (OR: 1.558; p = 0.027), mitral valve replacement (OR: 21.941, p < 0.001), supravalvular aortic stenosis repair (OR: 11.868, p = 0.016), and preoperative CPR (OR: 2.037, p = 0.001) (Table 4a). For mortality following thrombus formation, preoperative CPR (OR: 7.778, p < 0.001) was identified as an independent risk factor (Table 4b). Kaplan-Meier survival analysis revealed significantly reduced transplant-free survival in patients with thrombus formation (log-rank p = 0.004), with early separation of survival curves that persisted throughout the follow-up period (Fig. 3).

**Table 4a:** Risk factors for thrombus in neonates and early infants following congenital heart surgery.

Variable	Univariable			Multivariable		
	OR	95 % CI	p-Value	OR	95 % CI	p-Value
Age at OP (days)	0.997	0.990–1.003	0.286			
Weight at OP (kg)	0.828	0.621–1.105	0.200			
OP in neonate	1.240	0.867–1.774	0.239			
Tricuspid valve repair	5.234	2.004–13.670	**<0.001**	6.206	2.294–16.787	**<0.001**
ASO + VSD + Aortic arch	2.408	0.695–8.348	0.166			
CAVSD repair	1.979	0.682–5.737	0.209			
Rastelli procedure	1.826	0.412–8.100	0.428			
Norwood-type procedure	1.656	1.144–2.396	**0.007**	1.558	1.052–2.308	**0.027**
Mitral valve replacement	19.392	3.218–116.860	**0.001**	21.941	3.592–134.043	**<0.001**
Supra AS repair	6.420	1.167–35.304	**0.033**	11.868	1.597–88.178	**0.016**
Aortic vale replacement	3.191	0.355–28.708	0.301			
TAC + IAA	2.551	0.296–21.964	0.394			
Ross-Konno	2.125	0.254–17.755	0.486			
Heterotaxy	0.501	0.121–2.081	0.341			
Pre CPR	2.157	1.407–3.309	**<0.001**	2.037	1.316–3.154	**0.001**
Pre Cathecholamine	1.922	1.024–3.608	**0.042**			
STAT (4–5 vs 1–3)	1.136	0.828–1.558	0.429			
CPB time (minutes)	1.004	1.001–1.006	**0.003**	1.003	1.000–1.005	**0.050**

**Table 4b:** Risk factors for mortality after thrombus formation.

Variable	Univariable			Multivariable		
	OR	95 % CI	p-Value	OR	95 % CI	p-Value
Age at OP	0.999	0.982–1.016	0.906			
Weight at OP	0.447	0.203–0.985	**0.046**			
OP in neonate	1.078	0.425–2.734	0.875			
Tricuspid valve repair	0.971	0.109–8.639	0.979			
ASO + VSD + Aortic arch	10.370	0.908–118.451	0.060			
CAVSD repair	1.643	0.165–16.375	0.672			
Norwood procedure	1.868	0.788–4.428	0.156			
Mitral valve replacement	2.482	0.218–28.323	0.464			
CPR	8.467	3.395–21.115	**<0.001**	7.778	2.214–27.329	**<0.001**

OR: Odds ratio, CI: Confidence interval, OP: operation, ASO: Arterial switch operation, VSD: Ventricular Septal Defect, CAVSD: Complete atrioventricular septal defect, AS: Aortic stenosis, TAC: Truncus arteriosus communis, IAA: Interrupted aortic arch, CPR: cardiopulmonary resuscitation, STAT: The Society of Thoracic Surgeons-European Association for Cardio-Thoracic Surgery, vs: versus, CPB: cardiopulmonary bypass.

## 4 Discussion

### 4.1 Summary of the results

This study identified thrombus formation in 170 of 2331 neonates and early infants (7.3 %) undergoing congenital heart surgery with CPB. Independent risk factors for thrombus formation included tricuspid valve repair, mitral valve replacement, Norwood procedure, and preoperative CPR, with patients experiencing thrombus formation requiring significantly prolonged hospitalization.

### 4.2 Incidence of thrombosis

Previous studies have reported thrombosis rates of 3.6–11 % following pediatric cardiac surgery, with infants <1 year, particularly <31 days, at highest risk [[9], [10], [11]]. Our incidence of 7.3 % falls within this reported range and likely reflects our focus on the high-risk population of neonates and early infants. The median time to detect thrombosis was POD 7, emphasizing the need for close monitoring during the initial recovery period.

### 4.3 Pathogenesis and risk factors for thrombosis

Thrombus formation in neonates and early infants results from complex pathophysiological mechanisms based on Virchow's triad (abnormal blood flow, altered blood composition, and vessel wall injury) [12]. Abnormal blood flow includes blood stasis in cyanotic CHD and artificial shunts (Blalock-Taussig shunt), which promote thrombosis. In cyanotic CHD, polycythemia and increased blood viscosity lead to microcirculatory stasis, creating a risk for disseminated intravascular coagulation development [13,14]. Altered blood composition is characterized by neonatal coagulation immaturity. Inadequate production of vitamin K-dependent coagulation factors (II, VII, IX, X) and anticoagulant factors (antithrombin, proteins C and S) results in unstable hemostatic balance [15]. In infants aged 0–3 months, multiple factor abnormalities coexist with platelet dysfunction, while elevated factor VIII and increased platelet microparticles indicate a hypercoagulable state [4,16]. CPB exacerbates these abnormalities, causing platelet dysfunction, dilution and consumption of coagulation factors, inflammatory responses, and endothelial dysfunction [17]. In neonates, the relatively large priming volume leads to a pronounced hematocrit reduction and dilutional effects on coagulation factors. Our study demonstrated significantly longer CPB times in the thrombosis group, with elevated APTT and decreased fibrinogen on POD 3, suggesting complex interactions between consumptive coagulopathy and anticoagulation therapy. Vessel wall injury involves neutrophil activation and vascular endothelial damage from cyanosis [18], as well as endothelial injury from central venous catheter placement, with catheter-related thrombosis occurring in >20 % of pediatric cardiac patients [19]. In addition to these pathophysiological factors, clinical risk factors identified in our study are also important. Preoperative CPR reflects underlying hemodynamic instability and coagulation cascade activation during resuscitation, while preoperative catecholamine administration suggests cardiac dysfunction and prothrombotic hemodynamic changes.

### 4.4 Risk factors for thrombosis and mortality

Multivariate analysis identified specific surgical risk factors for thrombosis. Tricuspid valve repair demonstrated the highest risk, likely attributable to altered hemodynamics and turbulent flow around the valve. The Norwood procedure also showed increased thrombotic risk, attributed to systemic-to-pulmonary artery shunt creation and associated hemodynamic changes. Mitral valve replacement exhibited the highest odds ratio but requires cautious interpretation due to limited case numbers. Notably, procedural complexity (STAT categories) was not independently associated with thrombus formation, suggesting that patient-specific factors rather than procedural complexity itself are the primary drivers of thrombotic risk. Thrombus location analysis revealed a predominance of venous thrombosis, with the superior and inferior vena cava being the most frequent sites. This suggests that venous stasis and central venous catheter-related factors are crucial in thrombogenesis. Regarding post-thrombotic mortality, only preoperative CPR remained an independent risk factor, emphasizing the prognostic importance of preoperative hemodynamic instability.

### 4.5 Future prospective

Based on our findings, several strategies may reduce thrombotic complications in this high-risk population. Risk-stratified anticoagulation protocols could be developed according to specific surgical procedures, with enhanced monitoring for high-risk operations such as tricuspid valve repair and Norwood procedures. Advanced imaging modalities, including contrast echocardiography, may improve early detection of thrombus formation, particularly given our median detection time of 7 days. The development of biomarkers for thrombotic risk stratification could enable personalized anticoagulation management. Point-of-care coagulation monitoring may optimize anticoagulation therapy by addressing the complex interactions between consumptive coagulopathy and anticoagulation observed in our study while maintaining adequate thromboprophylaxis and reducing bleeding complications.

### 4.6 Limitations

This study has several inherent limitations of a retrospective, single-center design. Reliance on transthoracic echocardiography for thrombus detection may have resulted in underdiagnosis of small or poorly visualized thrombi, as angiography was only employed in select cases with hemodynamic compromise. The 23-year study period encompasses significant evolution in surgical techniques, anticoagulation protocols, and postoperative management, potentially limiting generalizability to current practice. Despite stratified analysis by case volume, the heterogeneity of cardiac conditions and procedures made it challenging to establish condition-specific risk stratification. We could not analyze genetic factors, inflammatory markers, or detailed hemodynamic parameters that may contribute to thrombotic risk. Although all patients had central venous catheters with standardized anticoagulation until line removal, catheter-related thrombosis may represent an important component of thrombotic events in this population. Long-term follow-up data regarding neurodevelopmental outcomes or chronic complications related to thrombus formation were not available, limiting assessment of long-term clinical impact.

## 5 Conclusions

Thrombus formation occurred in 7.3 % of neonates and early infants undergoing cardiac surgery with cardiopulmonary bypass, with tricuspid valve repair, Norwood procedure, and preoperative cardiopulmonary resuscitation identified as independent risk factors. Patients with thrombosis experienced prolonged hospitalization and significantly reduced long-term transplant-free survival, emphasizing the need for enhanced monitoring and risk-stratified anticoagulation protocols in this high-risk population.

## CRediT authorship contribution statement

**Muneaki Matsubara:** Writing – original draft, Methodology, Investigation, Formal analysis, Data curation. **Alessandra Poppe:** Writing – original draft, Investigation, Data curation. **Thibault Schaeffer:** Writing – review & editing. **Jonas Palm:** Writing – review & editing, Methodology, Investigation, Data curation. **Teresa Lemmen:** Writing – review & editing, Data curation. **Paul Philipp Heinisch:** Writing – review & editing. **Nicole Piber:** Writing – review & editing. **Andrea Amici:** Writing – review & editing. **Alfred Hager:** Writing – review & editing, Validation, Supervision, Investigation, Conceptualization. **Peter Ewert:** Writing – review & editing, Validation, Supervision, Methodology, Investigation, Conceptualization. **Jürgen Hörer:** Writing – review & editing, Validation, Supervision, Project administration, Conceptualization. **Masamichi Ono:** Writing – review & editing, Visualization, Supervision, Project administration, Investigation, Data curation.

## Funding statement

This study was supported by grants from the Förderverein des Deutschen Herzzentrums München.

## Declaration of competing interest

The authors declare that they have no known competing financial interests or personal relationships that could have appeared to influence the work reported in this paper.
